# Improving Antibiotic Stewardship for Diarrheal Disease With Probability-Based Electronic Clinical Decision Support: A Randomized Crossover Trial

**DOI:** 10.1001/jamapediatrics.2022.2535

**Published:** 2022-08-29

**Authors:** Eric J. Nelson, Ashraful I. Khan, Adama Mamby Keita, Ben J. Brintz, Youssouf Keita, Doh Sanogo, Md Taufiqul Islam, Zahid Hasan Khan, Md Mahbubur Rashid, Dilruba Nasrin, Melissa H. Watt, Sharia M. Ahmed, Ben Haaland, Andrew T. Pavia, Adam C. Levine, Dennis L. Chao, Karen L. Kotloff, Firdausi Qadri, Samba O. Sow, Daniel T. Leung

**Affiliations:** 1 Departments of Pediatrics and Environmental and Global Health, Emerging Pathogens Institute, University of Florida, Gainesville; 2 International Centre for Diarrhoeal Disease Research, Bangladesh, Dhaka, Bangladesh; 3 Center for Vaccine Development—Mali, Bamako, Mali; 4 Division of Epidemiology, University of Utah School of Medicine, Salt Lake City; 5 Center for Vaccine Development and the Department of Pediatrics, University of Maryland, Baltimore; 6 Department of Population Health Sciences, University of Utah School of Medicine, Salt Lake City; 7 Division of Infectious Diseases, University of Utah School of Medicine, Salt Lake City; 8 Division of Pediatrics Infectious Diseases, University of Utah School of Medicine, Salt Lake City; 9 Department of Emergency Medicine, Warren Alpert Medical School of Brown University, Providence, Rhode Island; 10 Institute for Disease Modeling, Bill & Melinda Gates Foundation, Seattle, Washington

## Abstract

**Importance:**

Inappropriate use of antibiotics for diarrheal illness can result in adverse effects and increase in antimicrobial resistance.

**Objective:**

To determine whether the diarrheal etiology prediction (DEP) algorithm, which uses patient-specific and location-specific features to estimate the probability that diarrhea etiology is exclusively viral, impacts antibiotic prescriptions in patients with acute diarrhea.

**Design, Setting, and Participants:**

A randomized crossover study was conducted to evaluate the DEP incorporated into a smartphone-based electronic clinical decision-support (eCDS) tool. The DEP calculated the probability of viral etiology of diarrhea, based on dynamic patient-specific and location-specific features. Physicians were randomized in the first 4-week study period to the intervention arm (eCDS with the DEP) or control arm (eCDS without the DEP), followed by a 1-week washout period before a subsequent 4-week crossover period. The study was conducted at 3 sites in Bangladesh from November 17, 2021, to January 21, 2021, and at 4 sites in Mali from January 6, 2021, to March 5, 2021. Eligible physicians were those who treated children with diarrhea. Eligible patients were children between ages 2 and 59 months with acute diarrhea and household access to a cell phone for follow-up.

**Interventions:**

Use of the eCDS with the DEP (intervention arm) vs use of the eCDS without the DEP (control arm).

**Main Outcomes and Measures:**

The primary outcome was the proportion of children prescribed an antibiotic.

**Results:**

A total of 30 physician participants and 941 patient participants (57.1% male; median [IQR] age, 12 [8-18] months) were enrolled. There was no evidence of a difference in the proportion of children prescribed antibiotics by physicians using the DEP (risk difference [RD], −4.2%; 95% CI, −10.7% to 1.0%). In a post hoc analysis that accounted for the predicted probability of a viral-only etiology, there was a statistically significant difference in risk of antibiotic prescription between the DEP and control arms (RD, −0.056; 95% CI, −0.128 to −0.01). No known adverse effects of the DEP were detected at 10-day postdischarge.

**Conclusions and Relevance:**

Use of a tool that provides an estimate of etiological likelihood did not result in a significant change in overall antibiotic prescriptions. Post hoc analysis suggests that a higher predicted probability of viral etiology was linked to reductions in antibiotic use.

**Trial Registration:**

Clinicaltrials.gov Identifier: NCT04602676

## Key Points

**Question:**

Improving antibiotic stewardship in clinical settings prone to inappropriate antibiotic use has been a persistent and difficult challenge, especially for diarrheal disease management.

**Findings:**

This study evaluated a decision support tool that uses an algorithm of patient-specific and location-specific features to estimate the probability a case of diarrhea is caused by a virus; use of the tool did not associate with a change in antibiotic use overall. When accounting for the probability that a case was viral, a reduction in antibiotic use was found.

**Meaning:**

Antibiotic stewardship for diarrheal disease can be aided by tools that predict viral causation.

## Teaser

This study attempts to determine whether the diarrheal etiology prediction algorithm impacts antibiotic prescriptions in patients with acute diarrhea.

## Introduction

Many children in low- and middle-income countries are exposed to antibiotics in early life, and antimicrobial treatment of diarrhea rarely follows recommendations of international guidelines.^1,2^ This problem persists despite World Health Organization (WHO) Integrated Management of Childhood Illness recommendations that antibiotics should only be given for bloody diarrhea or suspicion for cholera,^3^ and that most cases of pediatric diarrhea, especially in those younger than 24 months, are likely attributable to causes that are not responsive to antibiotics.^4^ The clinical environments in these contexts offer mixed incentives to adhere to guidelines regarding antibiotic prescribing.^5^ Qualitative research suggests an unexplored willingness among physicians to follow guidelines, and electronic clinical decision support (eCDS), by their ability to include real-time calculation of patient- and location-specific inputs, may represent an opportunity to improve guideline adherence.^5,6^

Our team previously developed a diarrheal etiologic prediction (DEP) algorithm, based on a modular aggregation of statistical models from a large multicenter study of pediatric diarrhea to predict the probability that a patient has a viral etiology of diarrheal illness.^7,8^ The DEP draws upon data from clinical history and symptoms of the patient (patient-specific sources) and location-specific sources, such as clinical presentation of prior patients, historical prevalence, and weather parameters.^7,9^ The performance of the DEP algorithm was assessed in an external validation study at sites in Mali and Bangladesh. We used a molecular diagnostic on stool samples as a gold standard to show a discriminatory performance area under the curve = 0.75, calibration-in-the-large α = −0.393, and calibration slope β = 1.287.^7,9^ The discriminative performance was comparable with that achieved in external validation of other commonly used prediction models for etiology of infection.^10,11^ In the current study, we incorporated the DEP algorithm into a smartphone-based eCDS tool for physicians to use in clinical encounters with pediatric patients with diarrhea and make informed decisions about antibiotic use. In this randomized crossover study, our primary objective was to test the hypothesis that the proportion of patient encounters with associated antibiotic use would be lower when physicians had access to the DEP compared with those without DEP access, without compromising clinical outcomes.

## Methods

### Study Design and Study Sites

We conducted a randomized crossover study at 3 government hospitals in Bangladesh and 4 government health care facilities in Mali (trial protocols are available in Supplements 1 and 2; eFigure 1 in Supplement 3). Research teams were from the Centre Pour Les Vaccins en Developpement, Mali, the Institute for Epidemiology, Disease Control and Research at the Bangladesh Ministry of Health and Family Welfare, and the International Centre for Diarrhoeal Disease Research, Bangladesh. University of Utah (135830), International Centre for Diarrhoeal Disease Research, Bangladesh (20003), and University Of Science Technical And Technologies of Bamako in Mali (2020/122/CE/FMOS/FAPH) provided institutional review board and ethics committee approval. Written informed consent was obtained from physicians and the parent/guardian of each child participant. A data safety monitoring plan and board were established a priori in the event of serious adverse events. This study adhered to the Consolidated Standards of Reporting Trials (CONSORT) reporting guidelines and was registered at ClinicalTrials.gov (NCT04602676P).

### Participants

Physician participants were recruited from pools of existing physicians; no incentive was provided. Eligible physicians were physicians who treated children with diarrhea and expected to remain at their site for the study period. The study was conducted without interruption of routine clinical workflow. Children aged 2 to 59 months with acute (<7 days) diarrhea (3 or more loose stools in the prior 24 hours) and household access to a cell phone for a 10-day follow-up call were eligible for enrollment. Patients were excluded if they had severe pneumonia, severe sepsis, meningitis, or other conditions aside from gastroenteritis, or mid-upper arm circumference, indicating severe malnutrition (≤115 mm if >6 months old, or ≤110 mm if 2-6 months old). The duration of follow-up for patient participants was 10 days.

Patient participant recruitment occurred at the time of presentation to the health care facility during daytime hours, regardless of subsequent hospitalization status; no incentive for patient participant was provided. Participants were screened by study staff, and data were recorded on a screening form. Physicians used the assigned eCDS for all pediatric diarrheal patients enrolled.

### Procedures

#### Intervention and Randomization

This was a randomized crossover study with a permuted block randomization scheme, stratified by site, that was developed using a random number generator for physician assignment with block sizes of 6 for the 3 Bangladesh sites and block sizes of 4 for the 4 Mali sites. In Bangladesh, physicians within hospitals were randomized according to permuted blocks of size 6. However, only the first 5 assignments within each hospital were used since each hospital had 5 participating physicians. Physician participants were assigned to either a control arm of an eCDS without the DEP (comparison, described below) or intervention arm of an eCDS with the DEP (intervention, described below) for the initial 4-week period (period 1), followed by a 1-week washout period before the 4-week crossover period (period 2). The research team set the DEP on vs off within study periods, depending on the electronically randomized assignment of the physician established before the start of the study. Patient participants were blinded to the intervention. Prior to study initiation, physicians received training on WHO guidelines for diarrheal management (eMethods in the Supplement 3) and were introduced to eCDS. No further guidance regarding antimicrobial use was provided.

### Comparison

The DEP was integrated into a previously developed Rehydration Calculator eCDS^2,12^ that digitized the WHO Integrated Management of Childhood Illness guidelines for the management of diarrheal disease (eFigure 4 in the Supplement 3). The design of the input page captured age, gender, weight (measured or estimated), diarrhea type (bloody, watery), clinical signs of dehydration (general disposition, sunken eyes, thirst, skin pinch), and danger signs (eFigure 3A in the Supplement 3). The output page recommended the volume of rehydration fluids, information on danger signs, and etiology-independent medication recommendations (eg, zinc). The DEP set on provided the probability of viral etiology (eFigure 3B in Supplement 3).

### Intervention

The DEP is a dynamic predictive model for viral etiology of diarrhea that integrates multiple sources of data, including both patient-specific and location-specific factors, in a principled statistical framework using a post-test odds formulation.^7^ Its development, integration into a smartphone application, and external validation in Mali and Bangladesh have been detailed previously.^7^ The DEP predictive model integrating patient-specific factors alone was used at all sites. Additionally, location-specific models were used for the sites proximal to the Global Enteric Multicenter Study sites from which location-specific models were derived. A post hoc analysis demonstrated that location-specific and nonlocation-specific methods varied the estimation of viral etiology by up to 8% (absolute difference).

### Outcomes

The primary outcome was the proportion of children prescribed an antibiotic by the treating physician. Secondary outcomes included resolution of diarrheal symptoms at 10 days postdischarge. Patient and household information were recorded at admission, discharge, and the 10-day follow-up on a case report form. At admission, information included diarrheal symptoms, respiratory symptoms, and preadmission antibiotics. During admission, information included therapies administered (antibiotics, oral rehydration salts, zinc, intravenous fluids) and at discharge, status, and additional medications given were recorded. At 10-day follow-up, information included questions on mortality, diarrhea resolution, respiratory symptoms, and additional medical care or antibiotics received.

### Sample Size

To evaluate the hypothesis that the DEP in the eCDS software would decrease antibiotic prescriptions compared with the eCDS without the DEP, estimates for physician and participant enrollment were calculated while recognizing that enrollment was census-based, and the pool of physicians was limited. Based on findings from Bangladesh and Mali sites in the Global Enteric Multicenter Study,^13^ we assumed that 90% of children would be prescribed antibiotics in the control arm (without DEP), and 80% of children would be prescribed antibiotics in the intervention arm (with DEP). With within- and between-period correlations for each physician of 0.15, and a sample size of 360 patient participants from 15 physician participants in 2 periods with 12 patients per physician and period, the study would achieve more than 95% power to detect the treatment effect with a type I error (α) of 0.05. If the patient participant enrollment was reached prior to the predetermined study period, enrolment was determined a priori to continue until the end of the study period given that aspects of the study outcomes were dependent on the duration of physician use of the software.

### Statistical Analysis

To evaluate the primary outcome, we examined risk differences (RDs) from a generalized linear mixed effects models with a logit link. ^14^



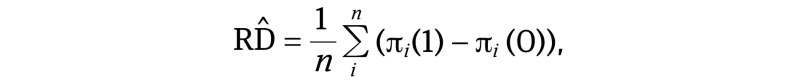



 We used parametric bootstrapping (5000 samples) to calculate 95% CIs for the RD estimate.^15^ The primary generalized linear mixed effects models included intervention (DEP), period, and their interaction with a random effect for site and physician nested in site to account for within-physician and within-site correlation. We also fit the same model with 2 additions (1) added an interaction term for arm and DEP-predicted probability of viral-only etiology in order to explore the expected change in behavior due to the value predicted from the DEP (including blinded value for the control arm), and (2) a random slope by physician to allow for a model fit with a different rate of behavior change by physicians. We did not include a DEP/control main effect. We assessed the presence of carryover effect using the treatment by period interaction term in each model. Data were merged from the 2 trials countries (each with unique institutional review board protocols). The level of significance was *P* = .05 and *P* values were 2-tailed. We conducted all analyses using R, version 4.1.0. (R Core Team).

## Results

**Figure 1.**
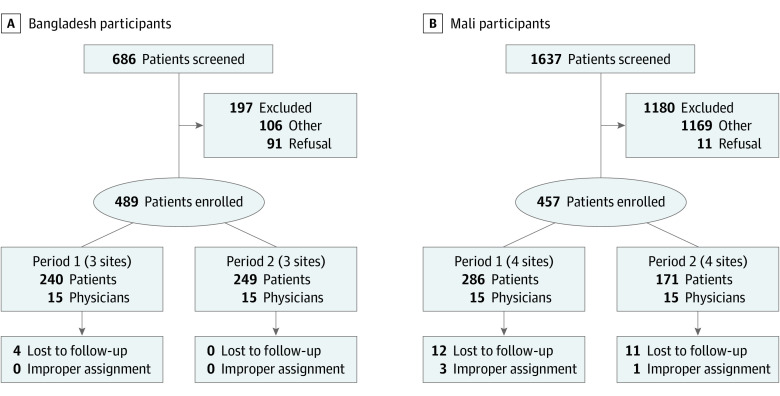
Enrollment Flow Diagram

A total of 30 physician participants and 946 patient participants were enrolled (Figure 1). In Bangladesh, from November 17, 2020, to January 21, 2021, 15 physicians and 489 patients were enrolled at the 3 sites (Figure 1A; eFigures 2 and 4 in Supplement 3) with 4 patients lost to follow-up. In Mali, from January 6, 2021, to March 5, 2021, 15 physicians and 457 patients were enrolled at the 4 sites (Figure 1B; eFigures 2 and 4 in Supplement 3) with 23 patients lost to follow-up; 4 cases were not analyzed because their recorded treatment/time period combinations were discrepant with the physicians' assigned treatment/time period combinations. Physicians in Bangladesh were 86.7% male and had a median age of 40 (IQR, 38.5-42.5) years. Physicians in Mali were 73.3% male and had a median age of 29 (IQR, 27-36) years. There was no physician attrition.

Among all patient participants, the enrollment rates were distributed evenly across the 2 study periods (eFigure 4 in Supplement 3) 57.1% were male, and the median age was 12 (IQR 8-18) months. Rates of severe dehydration were 0.22% in Mali and 11.1% in Bangladesh. Additional patient characteristics are provided (Table 1).

**Table 1.** Patient Participant Characteristics

Variable	No. (%)	
	DEP (n = 443)^a^	No DEP (n = 498)^a^
Sex		
Female	175 (39.5)	229 (46)
Male	268 (60.5)	269 (54)
Age, mo		
Mean (SD)	14.3 (9.2)	13.8 (9.2)
Median (IQR) (range)	12.0 (8.0-17.5) (2.0-54.0)	12.0 (8.0-18.0) (2.0-54.0)
Bloody diarrhea, yes	5 (1.1)	14 (2.8)
Vomit, yes	201 (45.4)	249 (50)
Breastfeeding		
Exclusive	57 (12.9)	69 (13.9)
No. (%)	89 (20.1)	92 (18.5)
Partial	297 (67)	337 (67.7)
MUAC		
Mean (SD)	13.8 (1.3)	13.9 (1.3)
Median (IQR) range	13.8 (13.0-14.5) (11.0-19.5)	14.0 (13.0-14.6) (11.3-19.0)
Antibiotic ordered, yes	309 (69.8)	381 (76.5)
Antibiotic ordered after discharge	4 (0.8)	7 (1.4)

Abbreviation: DEP, diarrheal etiology prediction; MUAC, mid-upper arm circumference.

^a^ Missing values: diarrhea resolution at 10 days 17 (DEP); 15 (no DEP).

### Primary Outcome

**Figure 2.**
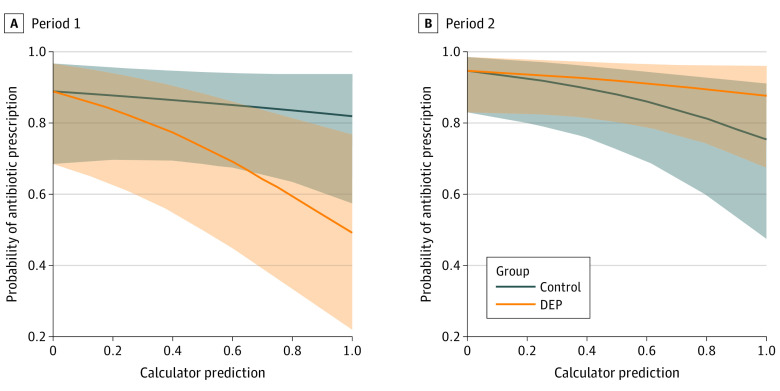
Fitted Probability of Antibiotic Prescription by the Diarrheal Etiology Prediction (DEP) Probability of Viral-only Etiology, by Period The dashed lines are the overall estimate for patients in each arm and does not account for site and physician random effects. The shaded portions indicate a 95% CI for each estimate.

Overall, 309 (69.8%) children in the DEP arm were prescribed antibiotics compared with 381 (76.5%) in the control arm. There was no statistically significant difference in the proportion of children prescribed antibiotics by physicians using the DEP (RD, −4.2%; 95% CI, −10.7% to 1.0%). A post hoc analysis was conducted to assess the primary outcome in the context of the predicted probability of a viral-only etiology. A statistically significant RD in antibiotic prescription was detected between the DEP and control arm (RD, −5.6%; 95% CI, −12.8% to −10%; Table 2). A 10% increase in predicted probability of viral-only diarrhea was associated with a 14% decrease in the odds of antibiotic prescribing (odds ratio, 0.86; 95% CI, 0.76-0.96) relative to the control arm (Figure 2; eTable in Supplement 3).

**Table 2.** Risk Differences for the Models Fit to Antibiotic Prescribing

Prescribed antibiotic	Period 1	Period 2	Full study
**Models adjusted for DEP assignment**			
RD (DEP minus no DEP)	−0.145	0.087	−0.042
95% CI (bootstrapped quantiles)	(−0.306 to −0.016)	(−0.028 to 0.207)	(−0.107 to 0.010)
**Models adjusted for DEP predicted values**			
RD (DEP minus no DEP)	−0.157	0.071	−0.056
95% CI (bootstrapped quantiles)	(−0.320 to −0.039)	(−0.038 to 0.179)	(−0.128 to −0.010)

Abbreviations: DEP, diarrheal etiology prediction; RD, risk difference.

We conducted post hoc subgroup analyses of the primary outcome by study period, study site, and physician. In the first study period, a significant decrease in the risk of antibiotic prescription was seen with DEP use (RD, −14.5%; 95% CI, −30.6% to −1.6%). In the second period, there was no difference in the risk of antibiotic prescription with and without DEP use (RD, 8.7%; 95% CI, −2.8% to 20.7%). When accounting for the predicted probability of a viral-only etiology, there was a significant risk difference in the first period (RD, −15.7%; 95% CI, −32.0% to −3.9%) but not in the second period (RD, 7.1%; 95% CI, −3.8% to 17.9%). In the second period, there was evidence of a carry-over effect, a 10% increase in predicted probability of viral-only diarrhea was associated with a 27% increase in the odds of antibiotic prescribing (odds ratio, 1.27; 95% CI, 1.01-1.59) relative to the control.

The effect of DEP among individual physicians was explored. We found that most physicians decreased antibiotic use as the DEP-predicted probability of viral-only increased, though some did not change behavior when using the DEP (eFigure 5 in Supplement 3). Physicians in Mali and Bangladesh decreased antibiotic use as the DEP-predicted probability of viral only increased during period 1, though this association was less pronounced in period 2 for both countries (eFigure 5 in Supplement 3). We found in multiple linear regression that physician age (continuous) was associated with the model estimated physician-level random slope effect (continuous, 0.038 (95% CI, 0.023-0.053; *P* = .05). There was no evidence of an association between sex or site with the physician-level random slope effect. Lastly, the study team estimated the post hoc power for the primary outcome; we used parametric bootstrapping to detect the treatment effect and the carry-over effect, assuming the effect sizes estimate in the primary model. The study team found that there was 67% power to detect the main treatment effect and 59% power to detect the carryover effect (alpha = 0.05).

### Secondary Outcomes

 Follow-up rates at 10 days postdischarge between the intervention and control arms were 96.2% and 97.2%, respectively (Table 3). There were no statistically significant differences in rates of diarrhea resolution at 10 days between the groups. Adverse events and severe adverse events were uncommon and could not be statistically estimated (Table 3).

**Table 3.** Clinical Course at Discharge and 10-Day Follow-up

Variable	No. (%)		Risk difference (95% CI)
	DEP (n = 443)^a^	No DEP (n = 498)^a^	
Able to contact, yes	426 (96.2)	484 (97.2)	−0.010 (−0.033 to 0.013)
Died after discharge, yes	1 (0.2)	1 (0.2)	0.000 (−0.006 to 0.006)
Diarrhea resolved at 10 d, yes	417 (97.9)	476 (98.6)	−0.007 (−0.024 to 0.011)
Days for diarrhea to resolve, median (IQR)	2 (1)	3 (1)	NA
Cough at 10 d, yes	47 (11.1)	60 (12.4)	−0.014 (−0.056 to 0.028)
Dyspnea at 10 d, yes	4 (0.9)	2 (0.4)	0.005 (−0.005 to 0.016)
Rhinorrhea at 10 d, yes	61 (14.3)	70 (14.5)	−0.002 (−0.048 to 0.044)

Abbreviation: DEP, diarrheal etiology prediction; NA, not applicable.

^a^ Missing values: died after discharge = 17 (DEP), 14 (no DEP); diarrhea resolution at 10 days: 15 (no DEP); 18 cough (DEP); 15 (no DEP); 17 difficulty breathing (DEP), 15 (no DEP); 17 runny nose (DEP), 15 (no DEP).

## Discussion

In this randomized crossover study, we did not demonstrate that the eCDS with the DEP led to a statistically significant reduction in antibiotic prescriptions by physicians. However, in post hoc analysis, physicians prescribed significantly fewer antibiotics in children who had higher predicted probability of viral diarrhea. These findings represent a technical and behavioral proof-of-concept that a probability-based eCDS in resource-limited settings can impact antibiotic use in pediatric patients.

The lack of a difference in antibiotic prescription between the study arms may have been due in part to lower power than estimated and unexpected differences between the 2 study periods. Our a priori power calculation relied on literature showing that approximately 90% of children with acute diarrhea with moderate to severe dehydration received antibiotics.^13^ We found antibiotic use was lower at 76.5% (control arm). This lower rate may be due to inclusion criteria that did not discriminate based on dehydration levels and/or a Hawthorne effect on physicians in the context of research cognizant that indications for antibiotic use are few for diarrheal illnesses. We additionally found that physician age was associated with the random slope for physician, suggesting that older physicians were less willing to change behavior based on the predicted value.

While most eCDS tools studied for antimicrobial stewardship provided antibiotic use recommendations, the eCDS with the DEP provided a probability estimate of viral etiology alone. The post hoc analyses suggest that providing physicians with an estimated probability of the etiology can improve appropriateness of antibiotic use. We saw overall an inverse association between probability of viral etiology and probability of antibiotic prescription. The probability of prescribing antibiotics was higher when the probability of a viral etiology was near 0 and decreased significantly as the probability of viral etiology rose to near 1.

Groupings of physicians in both countries were identified that did not change behavior in response to DEP use; these physicians were also those who had the highest rates of antibiotic use during the no-DEP period. These data suggest there are physician types that are more receptive and responsive to eCDS, and others that are more hesitant. This finding reveals a need to delineate these physician types, and to identify other modifiable factors that influence antibiotic prescribing.

Studies from low- and middle-income countries have identified the perceived threat to clinician autonomy and distrust of international guidelines lacking local context, as reasons for not using clinical decision support tools.^16,17,18^ Our approach leverages the ability of prediction algorithms to be updated in real-time, and provides clinicians with predictive analytics that include location-specific data. In parallel, this tool provides information for autonomous decision-making in the form of a probability estimate for disease etiology, rather than a set directive for antibiotic use. It is also possible that electronic tools may contribute toward the harnessing of a different Hawthorne effect where device interactions and perceived monitoring could influence prescribing behavior.^19^ Future studies focused on optimizing the output of such a tool for clinician use is warranted.

### Limitations

Our findings have several limitations. First, we enrolled a limited number of physician participants. While the number of physicians enrolled met the assumptions in the power calculation estimate, future studies with increased numbers of physicians in diverse clinical settings are needed and would also increase external validity. Second, we did not conduct diagnostic stool tests to verify etiologic predictions; however, the DEP algorithm was previously internally and externally validated at the same or similar study locations.^8,9^ Third, given that diarrheal disease-causing pathogens are often seasonal, and our study was conducted over a period of 9 weeks, it is possible that seasonality affected the study periods and that physicians were seeing patients with different characteristics between periods. However, after accounting for the predicted value of the DEP, there was no evidence for a period interacting with treatment effect. Fourth, physicians were not blinded. Fifth, we adjusted our block randomization approach in Bangladesh by dropping the last assignment. Given the small number of hospitals and physicians who participated in this study, there may have been advantages in using an adaptive randomization scheme, such as minimization that would directly target balancing features in the comparison arms. However, we did not find evidence to suggest a lack of balance between arms based on our truncated permuted block randomization approach.

## Conclusions

If replicated, the use of etiological prediction in decision support tools represents an important advancement to improve antibiotic stewardship in a clinical context prone to high rates of inappropriate antibiotic use. The results of this study represent a meaningful proof-of-concept for probability-based decision support to enable evidence-based antibiotic stewardship, especially in resource-limited settings.

## Notes

Supplement 1. eMethods. Assessment of dehydration and rehydration protocol eFigure 1. Trial locations and software localization eFigure 2. Study timeline eFigure 3. Rehydration calculator with the dep algorithm eFigure 4. Patient enrollment by location site and study period eFigure 5. Proportion of patients prescribed antibiotics eTable. Odds ratios for the models fit to antibiotic prescribing

Supplement 2. Bangladesh protocol

Supplement 3. Mali protocol

Supplement 4. Data sharing statement
