# Supplementary material for: Improving Antibiotic Stewardship for Diarrheal Disease With Probability-Based Electronic Clinical Decision Support: A Randomized Crossover Trial
Source: JAMA Pediatr. 2022 Aug 29;176(10):973–9. doi: 10.1001/jamapediatrics.2022.2535 (PMC9425282; doi:10.1001/jamapediatrics.2022.2535)
Supplement: Supplement 1. — eMethods. Assessment of dehydration and rehydration protocol eFigure 1. Trial locations and software localization eFigure 2. Study timeline eFigure 3. Rehydration calculator with the dep algorithm eFigure 4. Patient enrollment by location site and study period eFigure 5. Proportion of patients prescribed antibiotics eTable. Odds ratios for the models fit to antibiotic prescribing [file jamapediatr-e222535-s001.pdf]

Supplemental Online Content

Nelson EJ, Khan AI, Keita AM, et al. Improving antibiotic stewardship for diarrheal disease with probability-based electronic clinical decision support. *JAMA Pediatr.* Published online August 29, 2022. doi: 10.1001/jamapediatrics.2022.2535

eMethods. Assessment of dehydration and rehydration protocol

eFigure 1. Trial locations and software localization

eFigure 2. Study timeline

eFigure 3. Rehydration calculator with the dep algorithm

eFigure 4. Patient enrollment by location site and study period

eFigure 5. Proportion of patients prescribed antibiotics

eTable. Odds ratios for the models fit to antibiotic prescribing

This supplemental material has been provided by the authors to give readers additional information about their work.

© 2022 Nelson EJ et al. *JAMA Pediatrics*

eMethods. Assessment of dehydration and rehydration protocol

As summarized previously by Khan et al[1], the WHO assessment of dehydration uses four clinical features: general condition (well/alert, restless/irritable (<5 years only), lethargic/unconscious), eyes (normal/sunken), thirst (normal, drinks eagerly/ thirsty, not able to drink/ drinks poorly), skin pinch (goes back quickly in less than two seconds, slowly two to three seconds, very slowly greater than three seconds). These signs were scored 'No', 'Some', and 'Severe' to approximate 0-4%, 5-9%, and ≥10% weight loss, respectively, by scoring two features in the highest category. Patients with 'No' dehydration corrected ongoing losses with ORS, or 'Some' dehydration corrected current losses and ongoing losses with ORS; IV fluids were used for severe emesis or ileus. Patients with 'Severe' dehydration were rehydrated with IV fluids. IV regimen is 100ml/kg infused at 30ml/kg over one hour (< 1 year) or 30 minutes (>=1 year) followed by 70 ml/kg over five hours (< 1 year) or two and a half hours (>=1 year).

References:

1. Khan AI, Mack JA, Salimuzzaman M, et al. Electronic decision-support improves diarrhoeal disease guideline adherence (mHealth Diarrhoea Management, mHDM, Trial): a cluster randomized controlled trial. Lancet DH. May 2020;2:e250-258.

2. Rolla, Chisti M, Kache S, Smith J. Basic Paediatric Intensive Care in resource limited countries. In: Homer R, Walker I, Bell G, eds. Update in Anaesthesia. Special Edition ed. Singapore: COS Printers Pte Ltd; 2015:221-3.

3. Weil A, Chisti M, Harris J. Diarrheal Illness and Rehydration. In: Nelson B, Lee P, eds. Essential Clinical Global Health. 1st edition. ed: Sussex, John Wiley & Sons Ltd; 2015:104-13.

© 2022 Nelson EJ et al. *JAMA Pediatrics*

eFigure 1. Trial locations and software localization.

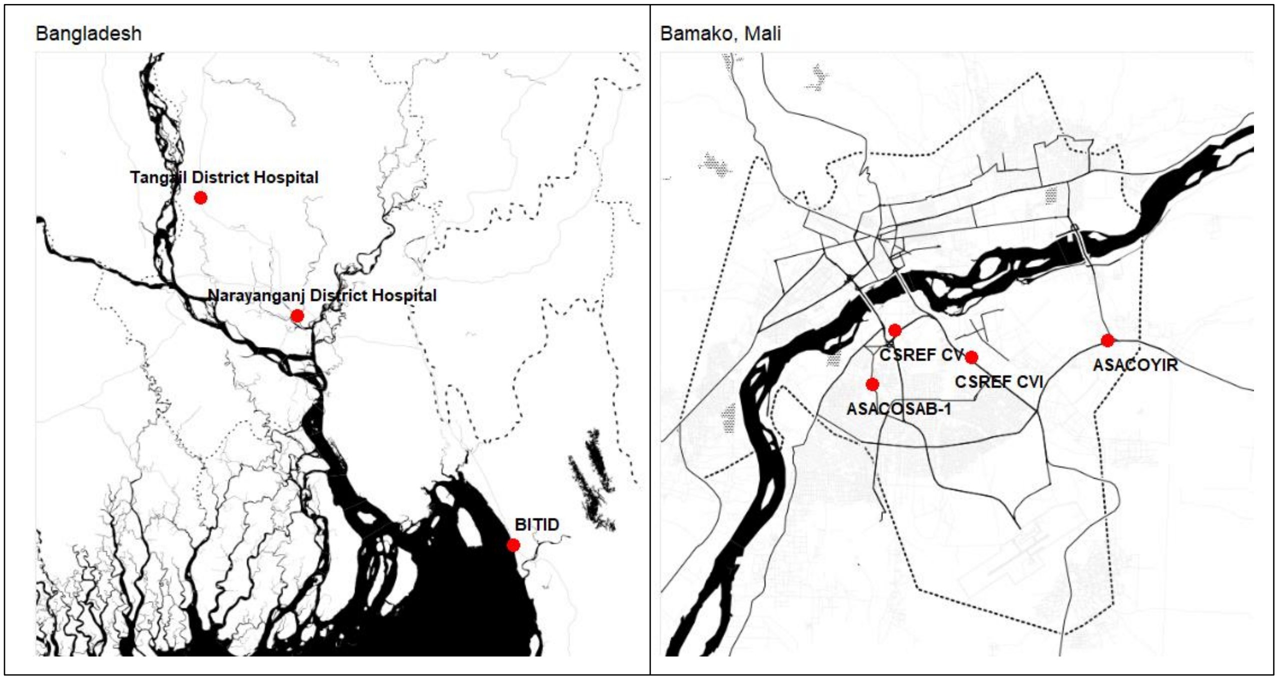

eFigure 1. Bangladesh and Mali Trial Locations. Red circles = study sites. The DEP determined which model(s) to use based on location. In Bangladesh, the DEP (at two sites localized to using seasonality, current patient, and weather models. One site in Bangladesh (Narayanganj District Hospital) and all sites in Mali used the current patient and seasonality models because these sites were near prior GEMS study sites and these two models performed best at GEMS sites. The software localizes to GEMS sites using cell tower ID numbers. If the tower of use is among the 5 towers closest to a GEMS site and is within a 200 km perimeter, the location of use is deemed a GEMS site.

eFigure 2. Study timeline.

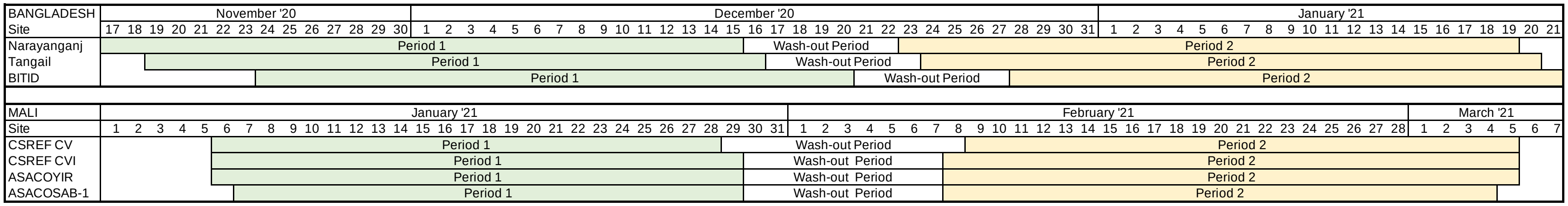

eFigure 2. Study timeline for periods and washouts by country and location.

© 2022 Nelson EJ et al. *JAMA Pediatrics*

eFigure 3. Rehydration calculator with the DEP algorithm

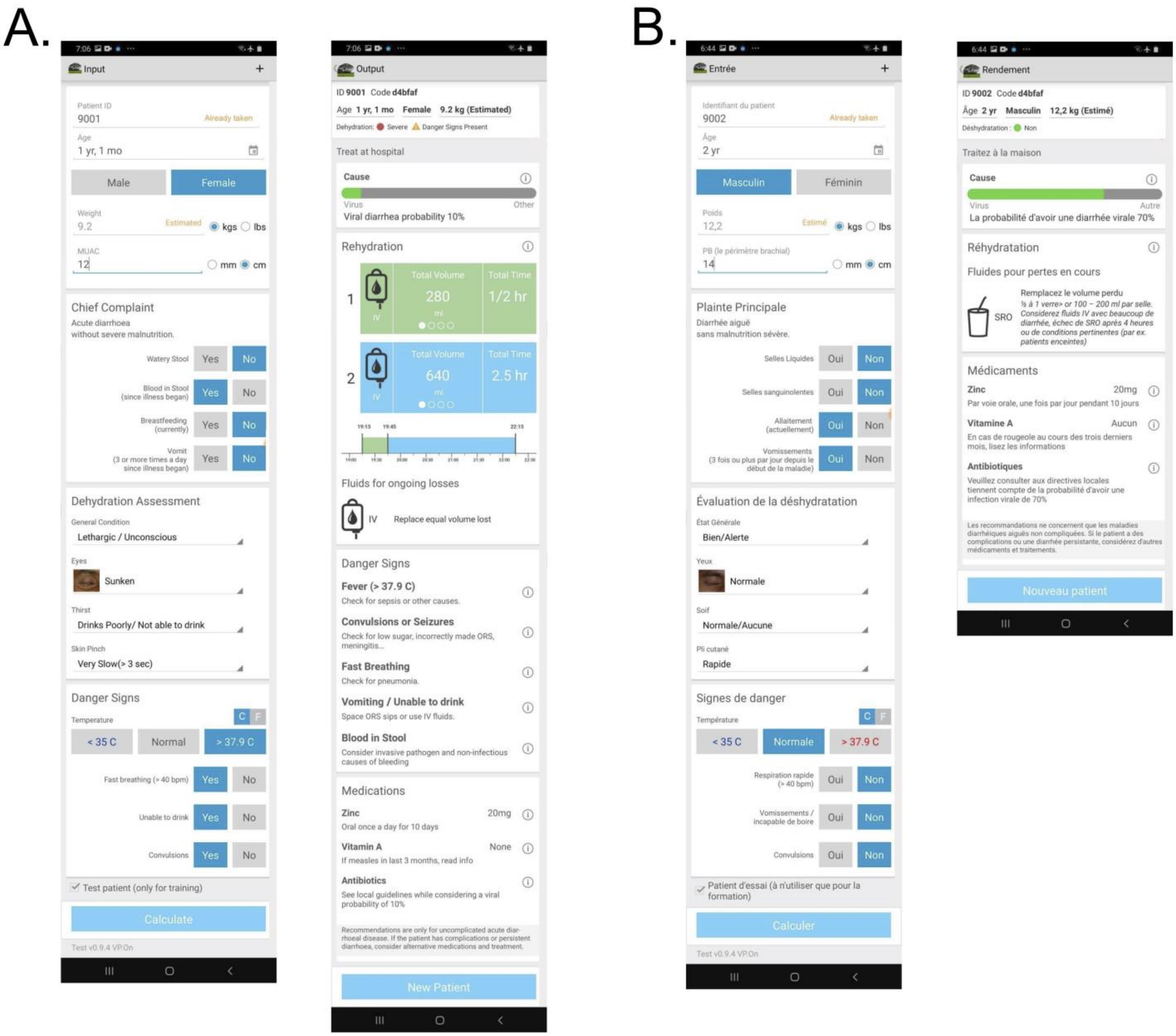

eFigure 3. Rehydration calculator with the DEP algorithm integrated. A. Severe dehydration (English); 10% probability of viral-only diarrhea. **B.** No dehydration (French); 70% probability of viral-only diarrhea. When DEP is 'off', the section titled 'cause' is not present.

© 2022 Nelson EJ et al. *JAMA Pediatrics*

eFigure 4. Patient enrollment by location site and study period

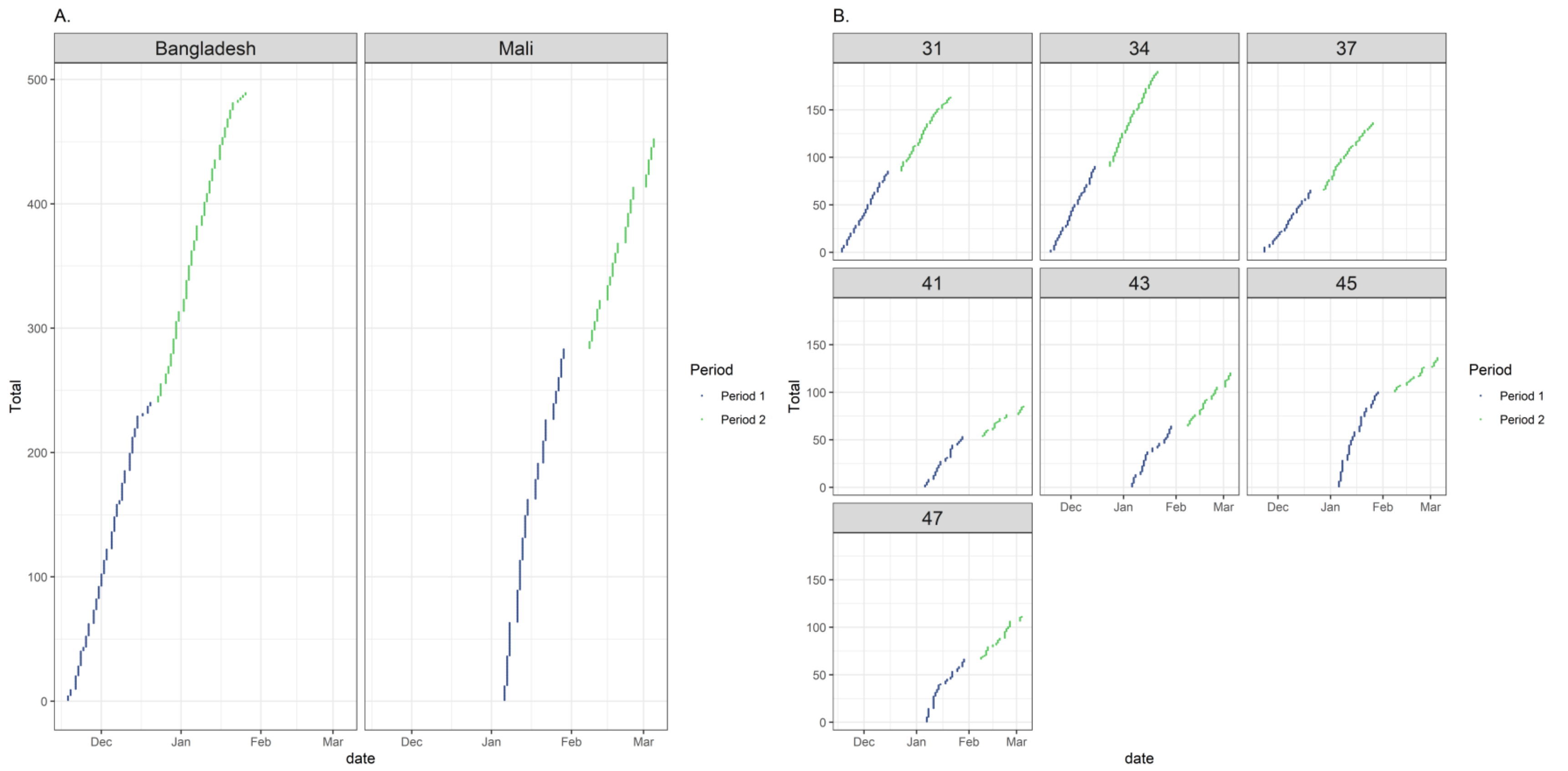

eFigure 4. Patient enrollment by location site and study period. A. Aggregate enrollment in Bangladesh (left) and Mali (right). **B.** Enrollment at each site in Bangladesh (31, 34, 37) and Mali (41, 43, 45, 47). Purple depicts the first study period and gold depicts the second study period.

© 2022 Nelson EJ et al. *JAMA Pediatrics*

eFigure 5. Proportion of patients prescribed antibiotics by provider, country, period.

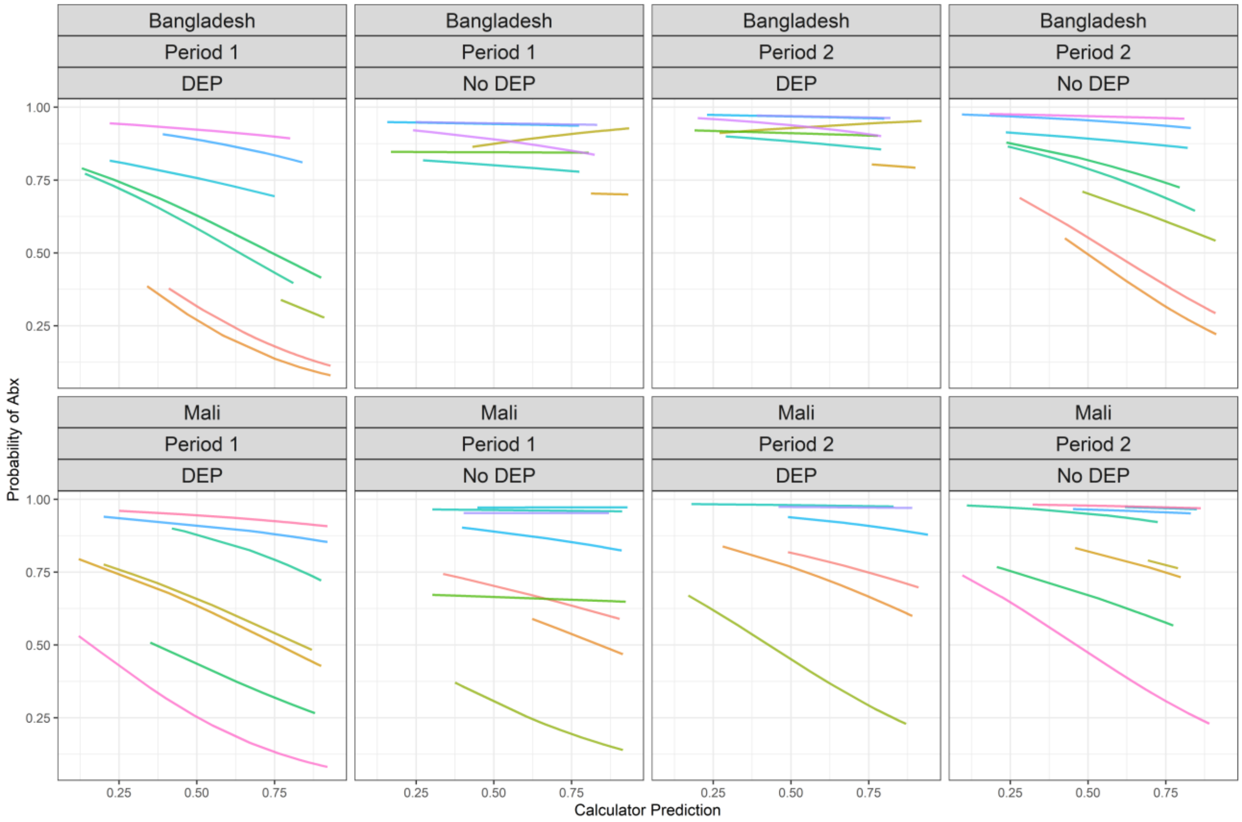

eFigure 5. Proportion of patients prescribed antibiotics by provider (all), country, and site. Fitted probability of antibiotic (Abx) prescription (Y axis) by the DEP calculator prediction of viral-only etiology (X axis). Panels are divided by country, study period, and DEP use. Colored lines represent individual provider estimates.

© 2022 Nelson EJ et al. *JAMA Pediatrics*

eTable. Odds Ratios (OR) for the models fit to antibiotic prescribing

Models adjusted for DEP assignment		
Prescribed Antibiotic	Period 1	Change from Period 1 to Period 2
OR (Change from no DEP to DEP)	0.37 (0.16, 0.87)	5.45 (1.08, 27.63)
P-value	0.023	0.041
**Model-adjusted Estimates for percent of patients prescribed antibiotic with and without DEP**		
	Period 1	Period 2
Percentage prescribed antibiotic with DEP	66% (40%, 85%)	91% (78%, 97%)
Percentage prescribed antibiotic without DEP	84% (64%, 94%)	84% (64%, 94%)
**Models adjusted for DEP predicted values**		
Prescribed Antibiotic	Period 1	Change from Period 1 to Period 2
OR per 10 percent increase in predicted probability of viral-only with no DEP	0.95 (0.81, 1.10)	0.89 (0.71, 1.11)
P-value	0.467	0.288
OR per 10 percent increase in predicted probability of viral-only (Change from no DEP to DEP)	0.86 (0.76, 0.96)	1.27 (1.01, 1.59)
P-value	0.011	0.037

© 2022 Nelson EJ et al. *JAMA Pediatrics*
