# Supplementary material for: Improving Antibiotic Stewardship for Diarrheal Disease With Probability-Based Electronic Clinical Decision Support: A Randomized Crossover Trial
Source: JAMA Pediatr. 2022 Aug 29;176(10):973–9. doi: 10.1001/jamapediatrics.2022.2535 (PMC9425282; doi:10.1001/jamapediatrics.2022.2535)
Supplement: Supplement 2. — Bangladesh protocol [file jamapediatr-e222535-s002.pdf]

**Principal Investigator:** Dr Ashraful Islam Khan	
**Research Protocol Title (BANGLADESH):** The acceptability and impact of Diarrheal Etiology Prediction (DEP) algorithm among physicians treating children with diarrhea in Bangladeshi hospitals.	

Background of the Project including Preliminary Observations:

Diarrheal diseases are a leading cause of morbidity and mortality in children worldwide, with an estimated one billion cases and 500,000 deaths annually. While the majority of deaths due to diarrhea occur in lower-income countries, infectious diarrhea remains a significant problem in high-income countries.

While the cornerstone of diarrheal disease management in children is rehydration, a number of other management decisions, including the use of antibiotics and laboratory testing, may impact the course of disease. Overuse of antibiotics may cause side-effects and lead to increased antimicrobial resistance in the community. Underuse of antimicrobials for some bacterial and protozoal pathogens may lead to prolonged duration of illness and facilitate transmission. Overuse of laboratory testing may have financial impact on both the patient and the healthcare system, and underuse may delay appropriate therapy or prevent recognition of outbreaks. Thus, accurate and cost-effective determination of diarrhea etiology is important for proper case management in children and for public health.

Paucity of decision-making tools and guidelines for management of pediatric diarrhea

Clinical prediction rules (CPRs) help clinicians interpret clinical information and can improve decision making. Given the lack of guidelines and effective clinical predictors, decisions for use of antibiotics and laboratory testing are mostly empiric in nature, based on a number of “rules of thumb” for which evidence is scant. Unfortunately, physician judgment does very poorly to predict both need for antibiotics and correct type of testing. A recent study of children presenting to Kenyan hospitals with diarrhea showed that reliance on dysentery as a proxy for *Shigella* infection led to the failure to diagnose shigellosis in nearly 90% of cases. Better tools for decision making and evidence-based guidelines regarding use of antibiotics and laboratory testing in children with diarrhea are clearly needed.

Decision-making for appropriate antibiotic use

The majority of decisions for use of antibiotics in diarrheal illnesses are made empirically and a large number (up to 70%) of patients with acute diarrhea are prescribed antibiotics. However, in contrast to high resource settings, bacterial pathogens may be very common in low resource settings. In both high and low resource settings, inappropriate use of antimicrobials leads to unnecessary toxicity for the individual, increased costs and an increase in antibiotic resistance in the community. Thus, methods for guiding appropriate use of antibiotics for pediatric diarrhea in both high- and low-resource settings are urgently needed.

Clinical prediction rules (CPRs) are decision-making rubrics that help clinicians estimate the likelihood of a patient outcome. A number of prominent prediction scores have been widely adopted for clinical use. Clinical prediction rules integrated into clinical decision-making have the ability to direct clinicians towards more evidence-based behaviors, resulting in improved care and reduction of costs. CPRs can also reduce antimicrobial usage, as shown by the use of scores for strep pharyngitis, and linking CPRs with testing guidance may further reduce antimicrobial usage. Thus, clinical prediction rules have the potential to help healthcare workers worldwide address clinical uncertainty and provide improved care for children with diarrhea.

We have recently use data from GEMS to derive a viral etiology prediction rule with an internal cross-validated area under the curve (AUC) of approximately 0.85. First, using data from GEMS, we trained a logistic regression model with viral etiology as dependent variable using the five most predictive clinical variables as independent variables. We then trained models with the same viral etiology response using both local climate and recent clinical trends as independent variables. We have now transferred the calculation of this prediction rule into a smart-phone application, called the Diarrhea Etiology Prediction (DEP) algorithm.

Hypothesis to be tested:

We hypothesize that Diarrheal Etiology Prediction (DEP) algorithm for calculating probability of viral etiology of diarrhea will reduce inappropriate antibiotic use among children <5 years old with acute diarrhea.

Specific Objectives:

Primary objective: To examine the acceptability and impact of a Diarrheal Etiology Prediction (DEP) among physicians treating children with diarrhea in Bangladeshi hospitals.

Research Design and Methods

Strategic Plan Overview: This is a randomized control trail crossover design, where clinicians will be randomized to periods where they will use a rehydration calculator application with or without the DEP. The crossover will include a washout period to reduce carryover effect. The study will be conducted over a 9-week period. We will use a random number generator to randomize clinicians to DEP or control arm for the first 4 weeks. After the first 4 weeks, there will be a 1-week washout period without decision support, after which each clinician will cross-over to the other arm for the next 4 weeks.

The DEP algorithm involves entry of clinical variables (age, bloody diarrhea, Breastfeeding, vomiting, mid-upper arm circumference- MUAC). The output provides the probability that the cause of the diarrhea is viral.

The DEP will be integrated as a feature into a Rehydration Calculator application that has previously been tested as part of two clinical studies in Bangladesh (icddr,b/ IEDCR; Khan AI et al., 2020 and Haque et al., 2017). The Rehydration Calculator with the DEP turned 'off' will serve as a control. The experimental arm of the study will have the DEP turned 'on'. The Rehydration Calculator is an electronic decision-support tool that provides recommendations for rehydration based on entry of age, gender, weight, and dehydration status. Given the outcome measures of this study, the Rehydration Calculator will be configured to not give antibiotic recommendations.

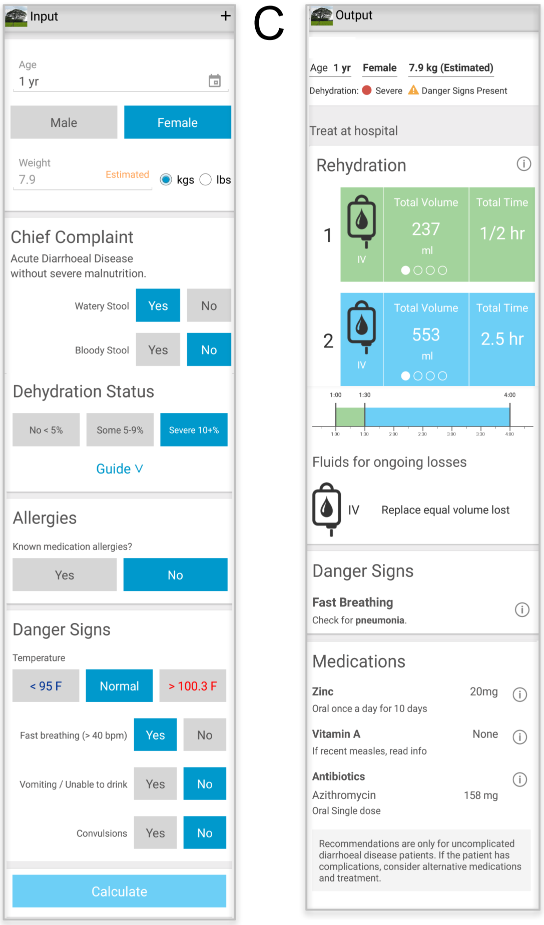

Fig 1. A. Data entry input page. **B.** Output page with recommendations for fluids, action items for danger signs, and medications. Educational information is available on each "i" tab.

Study Sites: 3 Hospitals in Bangladesh. The Sites are Narayanganj General (Victoria) Hospital, Tangail 250 Bed District Hospital, Bangladesh Institute of Tropical and Infectious Diseases (BITID) (Fig 3).

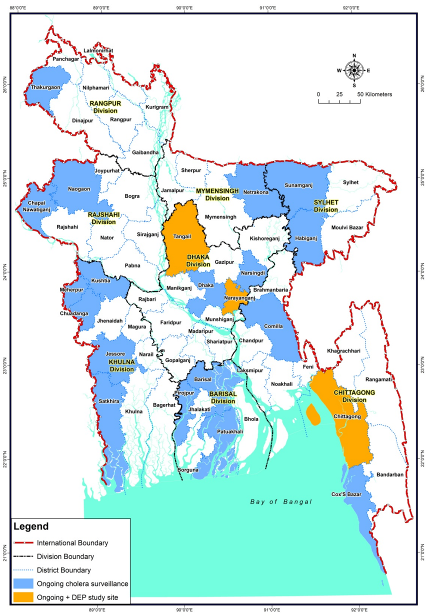

Fig 3: Study location: Of the 22 cholera surveillance sites, 2 district hospitals (Narayanganj and Tangail) and BITID, Chittagong.

Clinical setting:

The clinical approach will adhere to World Health Organization guidelines for the management of uncomplicated diarrheal disease in children with No, Some and Severe dehydration. These guidelines have previously been adapted to a digital format (e.g. Rehydration Calculator) and evaluated in a pilot and cluster randomized controlled trial (icddr,b/ IEDCR; Khan AI et al, submitted 2019 and Haque et al. PLoS NTD 2017); clinically relevant outcome measures in these studies were equivalent or improved with digital decision-support. The Rehydration Calculator version to be used in this study will include unchanged weights estimations and oral and intravenous (IV) fluid calculations;

changes will include elimination of the antibiotic recommendations and replacement with the viral diarrhea probability. Two district level hospitals and BITID will be selected along with about 15 physicians after fulfilling some criteria.

DEP Application

The DEP algorithm will be integrated as a feature into a Rehydration Calculator application that has previously been tested as part of two clinical studies in Bangladesh (icddr,b/ IEDCR; Khan AI et al, 2020) and Haque et al. PLoS NTD 2017). The Rehydration Calculator with the DEP turned 'off' will serve as a control. The experimental arm of the study will have the DEP turned 'on' (Fig 2). The Rehydration Calculator is an electronic decision support tool that provides recommendations for rehydration based on entry of age, gender, weight, and dehydration status. Given the outcome measures of this study, the Rehydration Calculator will be configured to not give antibiotic recommendations.

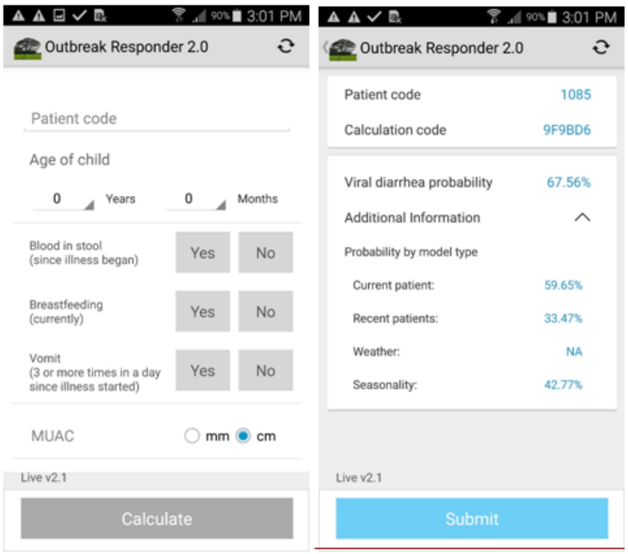

Fig 2: Sample screenshot of DEP mobile application on a Samsung Galaxy smart-phone.

a. Detailed Research Methods

c.i. **Study design.**

This is a randomized crossover study, where clinicians will be randomized to periods where they will use a rehydration calculator application with or without the DEP algorithm. The crossover will include a washout period to reduce carryover effect. The study will be conducted over a 9-week period. We will use a random number generator to randomize clinicians to DEP (use of the etiology calculator) or control arm (use of a previously-tested rehydration calculator) within site for the first 4 weeks. After the first 4 weeks, there will be 1-week washout period without decision support, after which each clinician will cross-over to the other arm for the next 4 weeks.

This study will also use qualitative methods: In-depth Interviews with physicians and observation at health care facilities. There will be respondents from two groups: rehydration calculator with DEP and rehydration calculator without DEP. The qualitative field researcher will collect data from both the group from the selected 3 hospital sites. To address gender dimensions, the qualitative research team will employ a female field researcher.

c.ii. **Study population and enrolment:**

We will enrol both 1) diarrhea-treating clinicians, and 2) children presenting with acute diarrhea.
We will use the following inclusion/exclusion criteria for enrolling clinicians:

Inclusion criteria:
- Physician providing acute care for children with diarrhea at study hospitals
- Available to answer survey questionnaire

Exclusion criteria:
- Planning to leave the study site prior to completion of the research
- Inability to read English

We will utilize the following inclusion/exclusion criteria when enrolling children:

Inclusion criteria:
- Age 2-59 months
- Less than 7 days of diarrheal symptoms from onset to presentation
- Three or more loose stools in the prior 24 hours

- Access to use of a cell phone

Exclusion criteria:

- No parent or primary caretaker available for consent
- Diarrhea lasting longer than 7 days
- Severe pneumonia, severe sepsis, meningitis, or other condition aside from gastroenteritis
- MUAC of <=115 if >6 months, or <=110 if 2 to 6 months of age [we will exclude severely malnourished children because they would follow a different antibiotic protocol]

For the purpose of enrolment in our study, we will utilize the World Health Organization (WHO) definition for acute diarrhea, which is 3 or more loose stools per day in the last 24 hours. Subjects with chronic diarrhea (diarrhea lasting more than 7 days) will be excluded, as this study is focused on acute diarrhea.

Staff Training: Staff will receive intensive training regarding all study procedures prior to the study. A study coordinator will be trained to use the randomization block and distribute appropriate mobile phones to physicians and keep track of the devices. The study coordinator will also be in charge of administering questionnaires to physicians (details described in data collection section).

Staff will enrol patients according to the inclusion/exclusion criteria and they will receive training which includes an in-depth review of how to appropriately assess symptoms and MUAC. They will also be trained in calling patients for a post-visit follow up questionnaire.

Doctors will receive training on how to use the Rehydration Calculator and DEP application prior to the study. They will complete questionnaires before and after the study regarding previous use of clinical calculators, access to internet and willingness to use a new app that will help to predict etiology of diarrhea.

Enrolment and consent of physicians

At each site, we will recruit clinicians whose responsibilities include the care of paediatric diarrhea patients. Study staff will determine if the clinician is eligible and then provide detailed information of the study. Once the clinician exhibits understanding, written consent will be obtained. There will be two questionnaires administered- before and after the study period. The post-study questionnaire will also include a brief focused interview. Only clinicians who have completed the entire study will be eligible for this questionnaire.

Enrolment and consent of patients

Study staff will be situated in the intake areas and will identify children 2–59 months with diarrheal illness. Study staff will select consecutive subjects between 8am and 2pm on weekdays for enrolment on arrival to the Hospital. Potential participants' information (age and time of intake, etc.) will be recorded in a screening log. Study staff and nurses will inform parents or primary caretakers (PPC) of patients who meet the inclusion/exclusion criteria about the study. If PPC is interested in the study, study staff will determine if the child is eligible and then provide more detailed information to the PPC's of eligible subjects. Detailed information will be provided in Bangla and will include goals, risks, and benefits of the study. Once the PPC exhibits understanding, written consent will be obtained in the local language prior to enrolling the subject in the study. Illiterate PPC's will give consent by marking the consent form with their thumbprint or signature after a study staff member reads the consent form in Bangla. Children without a PPC present will be excluded from the study. The last signature on the consent will be from the enrolling staff member.

c.iii. Data collection

Clinicians: We will use a screening form (Appendix 3) and questionnaire (Pre- and post-Study) to collect quantitative (Appendix 4 & 5) data regarding clinician attitudes and experiences using the App and its role in diarrheal management. These questionnaires will be administered by study coordinators at 2 time points: 1) at the start of the study, and 2) at the end of the 2nd 4 weeks. We will also use the App itself to collect data regarding physician usage.

Children: For each enrolled patient, study staff will collect:

- At enrolment, demographic and clinical variables

- At discharge, hospital outcome measures, including intravenous and oral fluids, and antibiotics, zinc administered
- At 10-days post-discharge, health outcomes including persistence of diarrheal and abdominal symptoms, returns to healthcare, and antibiotic use since discharge, death information will be collected over phone call. We will perform 2 attempts through phone call to reach every participant for 10 days post discharge interview. If the participants are not available through these phone call, we will go for next participant.

Instruments: As mentioned, electronic tools that are compliant to the protections of PHI (Protected Health Information) will be used in this study. These include the Outbreak Responder software used by Dr. Khan et al., 2020 in the prior mHDM cluster randomized controlled trial (documentation available at www.outbreakresponder.org), and if needed, RedCap. Outbreak Responder was used successfully by icddr,b teams in two previous studies in collaboration with Dr. E. Nelson (UF).

Data Storage: Data collected on Outbreak Responder software will be stored on a secure cloud-based server (server is physically based in Singapore; this is a HIPAA compliant server managed by Amazon Web Services) and locally on a secure icddr,b server. Access to the server(s) will be restricted and the server management conforms to industry standard for health information.

Sample Size Calculation and Outcome (Primary and Secondary) Variable(s)

For estimated 90% of children receiving antibiotics in control arm (per mHDM study; Khan AI, et al, in review at *Lancet Digital Health*), and estimated 80% of children receiving antibiotics in DEP arm, along with a within- and between- period correlations for each physician of 0.15, a sample size of 360 children (15 clinicians, with 2 cross-over periods and 12 patients during each period) will achieve a power above 95% to detect a 2-sided significance of 0.05. If enrolment is reached prior to the pre-determined study period, enrolment will be continued until the end of the study period; this is important given that aspects of the study outcomes are dependent on weeks of use.

Data Analysis/ primary endpoint (outcome)

The primary endpoint of estimating the proportion difference of children given antibiotic prescriptions in the control versus the DEP arms can be analysed using a generalized linear mixed model (GLMM). The response is binary (yes or no), and we will treat provider and clinic as random, while accounting for the time period in order to estimate the treatment effect. All data analyses will be conducted in R. For the physician satisfaction end point, we will use characteristic tables to summarize the results of the survey.

Definitions of Serious Adverse Events (SAE) and Adverse Events:

Serious Adverse Event: Any adverse event that results any of the following outcomes: death, significant disability/incapacity, and/or life-threatening situation of any patient within the study period (enrolment of patient at admission to 10 days after discharge).

Adverse Events: An adverse event will be defined as an untoward medical event (e.g. new onset of vomiting, convulsion, shock, acute renal failure, over-hydration and electrolyte imbalance) temporally associated with a medical intervention in a patient that does not necessarily have a causal relationship with the treatment. For the adverse events we will follow the clinical judgements of hospitals physicians and their advice for lab investigations.
