# Supplementary material for: Improving Antibiotic Stewardship for Diarrheal Disease With Probability-Based Electronic Clinical Decision Support: A Randomized Crossover Trial
Source: JAMA Pediatr. 2022 Aug 29;176(10):973–9. doi: 10.1001/jamapediatrics.2022.2535 (PMC9425282; doi:10.1001/jamapediatrics.2022.2535)
Supplement: Supplement 3. — Mali protocol [file jamapediatr-e222535-s003.pdf]

Protocol Title (MALI): The acceptability and impact of Diarrheal Etiology Prediction (DEP) algorithm among physicians treating children with diarrhea

Version Number: 1.0
Version Date: 09 June 2020

Sponsor: University of Utah

Investigators:

University of Utah

Daniel T. Leung, MD, MSC (Protocol Principal Investigator)

University of Maryland School of Medicine

Dilruba Nasrin, MBBS, PhD

CVD-Mali (Bamako, MALI)

Samba Sow, MD, MS (Site Principal Investigateur)
Adama Mamby Keita, MD
Doh Sanogo, MD
Youssouf Keita, MD
Fadima Cheick Haidara, MD

List of Abbreviations:

AUC	Internal cross-validated area under the curve
CHADS2 Score	Congestive heart failure, High blood pressure, Age, Diabetes, Stroke
CPR	Clinical prediction rules
CURB-65 Score	Confusion, Urea, Respiratory rate, Blood pressure, age 65 or older
CVD	Center for Vaccine Development
DEP	Diarrhoea Etiology Prediction algorithm
DSMP	Data and Safety Monitoring Plan
GEMS	Global Enteric Multicenter Study
HIPAA	Health Insurance Portability and Accountability Act
LMICS	Lower- and middle-income countries
MUAC	Mid-Upper Arm Circumference
NSTEMI	Non-ST-elevation Myocardial Infarction
PHI	Protected Health Information
PPC	Parent or primary caretaker
SAE	Serious Adverse Event
TAC	TaqMan Array Card
TIMI Score	Thrombolysis In Myocardial Infarction
VIDA	Vaccine Impact on Diarrhea in Africa
WHO	World Health Organization

Project Summary

Research Protocol Title: The acceptability and impact of Diarrheal Etiology Prediction (DEP) algorithm among physicians treating children with diarrhea

Background (brief):

a. Burden:

Diarrhoeal diseases are a leading cause of morbidity and mortality in children worldwide, with an estimated one billion cases and 500,000 deaths annually. While the majority of deaths due to diarrhoea occur in lower-income countries, infectious diarrhoea remains a significant problem in high-income countries. Accurate and cost-effective determination of diarrhoea etiology is important for proper case management in children and for public health.

b. Knowledge gap:

Clinical prediction rules (CPRs) help clinicians interpret clinical information and can improve decision making. A recent systematic review showed that out of 137 studies of conditions for which clinical prediction rules have been developed for children, only 2 were for diarrhoea, both of which are for the assessment of dehydration. Similarly, the majority of available guidelines for pediatric diarrhoea are focused on the route, timing, and choice of fluids for rehydration. However, these studies were limited by low rates of pathogen identification, small sample sizes, use of a single study site, and suboptimal prediction performance. Better tools for decision making about the diarrhea etiology and evidence-based guidelines regarding use of antibiotics and laboratory testing in children with diarrhoea are clearly needed.

c. Relevance:

The majority of decisions for use of antibiotics in diarrhoeal illnesses are made empirically. In lower and middle-income countries (LMICs), due to cost constraints, etiological diagnosis is rarely made, and a large number (up to 70%) of patients with acute diarrhoea are prescribed antibiotics. In both high and low resource settings, inappropriate use of antimicrobials leads to unnecessary toxicity for the individual, increased costs and an increase in antibiotic resistance in the community. Thus, methods for guiding appropriate use of antibiotics for pediatric diarrhoea in both high- and low-resource settings are urgently needed.

Clinical prediction rules (CPRs) are decision-making rubrics that help clinicians estimate the likelihood of a patient outcome. Clinical prediction rules integrated into clinical decision making have the ability to direct clinicians towards more evidence-based behaviors, resulting in improved care and reduction of costs and have the potential to help healthcare workers worldwide address clinical uncertainty and provide improved care for children with diarrhoea.

Hypothesis: We hypothesize that Diarrheal Etiology Prediction (DEP) algorithm for calculating probability of viral etiology of diarrhea will safely reduce inappropriate antibiotic use among children <5 years old with acute diarrhea.

Objectives: To examine the acceptability and impact of a Diarrheal Etiology Prediction (DEP) algorithm among physicians treating children with diarrhea.

Methods: This is a randomized crossover study and clinicians will be randomized to periods where they will use a rehydration calculator application with or without the DEP. The crossover will include a washout period to reduce carryover effect. The study will be conducted over a 9-weeks period. We will use a random number generator to randomize clinicians to DEP or control arm for the first 4 weeks. After the first 4 weeks, there will be a 1-week washout period without decision-support, after which each clinician will cross-over to the other arm for the next 4 weeks.

Outcome measures/variables:

The primary outcome is the proportion of children given antibiotic prescriptions, as assessed by hospital records. Secondary outcomes are the proportion of children with resolution of diarrheal symptoms at 10-days after enrollment, and clinician satisfaction towards use of the DEP, as assessed by pre- and post-study questionnaires

Description of the Research Project

Hypothesis to be tested: We hypothesize that Diarrheal Etiology Prediction (DEP) algorithm for calculating probability of viral etiology of diarrhea will safely reduce inappropriate antibiotic use among children <5 years old with acute diarrhea.

Primary objective: To examine the acceptability and impact of a Diarrheal Etiology Prediction (DEP) among physicians treating children with diarrhea.

Background of the Project including Preliminary Observations:

Diarrhoeal diseases are a leading cause of morbidity and mortality in children worldwide, with an estimated one billion cases and 500,000 deaths annually.[1] While the majority of deaths due to diarrhoea occur in lower-income countries, infectious diarrhoea remains a significant problem in high-income countries. Aside from the immediate morbidity, potential long-term sequelae of diarrhoea in children in low-resource settings include malnutrition, growth faltering, and deficits in cognitive development.[2]

While the cornerstone of diarrhoeal disease management in children is rehydration, a number of other management decisions, including the use of antibiotics and laboratory testing, may impact the course of disease.[3] Overuse of antibiotics may cause side-effects and lead to increased antimicrobial resistance in the community. Underuse of antimicrobials for some bacterial and protozoal pathogens may lead to prolonged duration of illness and facilitate transmission. This can result in increased days of school or parental work missed, and among malnourished children in resource-poor settings, potential for growth faltering or death. Overuse of laboratory testing may have financial impact on both the patient and the healthcare system, and underuse may delay appropriate therapy or prevent recognition of outbreaks. Thus, accurate and cost-effective determination of diarrhoea etiology is important for proper case management in children and for public health.

Paucity of decision-making tools and guidelines for management of pediatric diarrhoea

Clinical prediction rules (CPRs) help clinicians interpret clinical information and can improve decision making.[4, 5] A recent systematic review showed that out of 137 studies of conditions for which clinical prediction rules have been developed for children only 2 were for diarrhoea,[6] both of which are for the assessment of dehydration.[7, 8] Similarly, the majority of available guidelines for pediatric diarrhoea are focused on the route, timing, and choice of fluids for rehydration.[9-11] A few studies in the past 30 years studied the use of clinical predictors to estimate the probability of a bacterial cause of diarrhoea.[12-14] However, these studies were limited by low rates of pathogen identification, small sample sizes, use of a single study site, and suboptimal prediction performance.

Given the lack of guidelines and effective clinical predictors, decisions for use of antibiotics and laboratory testing are mostly empiric in nature, based on a number of "rules of thumb" for which evidence is scant. Unfortunately, physician judgment does very poorly to predict both need for antibiotics and correct type of testing. A recent study of children presenting to Kenyan hospitals with diarrhoea showed that reliance on dysentery as a proxy for *Shigella* infection led to the failure to diagnose Shigellosis in nearly 90% of cases.[15] Better tools for decision making and evidence-based guidelines regarding use of antibiotics and laboratory testing in children with diarrhoea are clearly needed.

Decision-making for appropriate antibiotic use

The majority of decisions for use of antibiotics in diarrhoeal illnesses are made empirically. In lower- and middle-income countries (LMICs), due to cost constraints, etiological diagnosis is rarely made, and a large number (up to 70%) of patients with acute diarrhoea are prescribed antibiotics.[16-18] However, in contrast to high resource settings, bacterial pathogens may be very common in low resource settings. In the multicenter Global Enteric Multicenter Study (GEMS) study, we found that detection of *Shigella* ranged from 16-78% of children with dysentery and 2-43% of children with watery diarrhoea.[19] In both high and low resource settings, inappropriate use of antimicrobials leads to unnecessary toxicity for the individual, increased costs and an increase in antibiotic resistance in the community. Thus, methods for guiding appropriate use of antibiotics for pediatric diarrhoea in both high- and low-resource settings are urgently needed.

Clinical prediction rules (CPRs) are decision-making rubrics that help clinicians estimate the likelihood of a patient outcome.[20] A number of prominent prediction scores have been widely adopted for clinical use. Examples include the CHADS2 score for stroke risk in patients with atrial fibrillation,[21] the TIMI score for mortality in patients with NSTEMI,[22] and the CURB-65 Score for mortality in community-acquired pneumonia.[23] Clinical prediction rules integrated into clinical decision-making have the ability to direct clinicians towards more evidence-based behaviors,[24] resulting in improved care and reduction of costs.[25] CPRs can also reduce antimicrobial usage, as shown by the use of scores for strep pharyngitis,[26] and linking CPRs with testing guidance may further reduce antimicrobial usage.[27] Thus, clinical prediction rules have the potential to help healthcare workers worldwide address clinical uncertainty and provide improved care for children with diarrhoea.

We have recently use data from GEMS to derive a viral etiology prediction rule with an internal cross-validated area under the curve (AUC) of approximately 0.85. We used a post-test odds formulation method, which takes into account odds from multiple models or tests. First, using data from GEMS, we trained a logistic regression model with viral etiology as dependent variable using the five most predictive clinical variables as independent variables. We then trained models with the same viral etiology response using both local climate and recent clinical trends as independent variables. For each model, odds of viral etiology versus other known aetiologies are generated by estimating the conditional distribution of training predictions using kernel density estimates. The odds generated for each model are multiplied along with a pre-test odds to determine an overall odds of a viral etiology. We have now transferred the calculation of this prediction rule into a smart-phone application, called the Diarrhoea Etiology Prediction (DEP) algorithm. We have used TAC data from the VIDA (Vaccine Impact on Diarrhea in Africa) study to externally validate the DEP algorithm's ability to predict viral etiology of diarrhea. In this application, our objective is to determine the acceptability and use of the DEP among clinicians caring for children with diarrhea.

Research Design and Methods

Strategic Plan Overview: This is a randomized crossover study, where clinicians will be randomized to periods where they will use a rehydration calculator application with or without the DEP. The crossover will include a washout period to reduce carryover effect. The study will be conducted over a 9-week period. We will use a random number generator to randomize clinicians to DEP or control arm for the first 4 weeks. After the first 4 weeks, there will be a 1-week washout period without decision support, after which each clinician will cross-over to the other arm for the next 4 weeks.

The DEP algorithm involves entry of clinical variables (age, bloody diarrhea, Breastfeeding, vomiting, mid-upper arm circumference- MUAC). The output provides the probability that the cause of the diarrhea is viral.

The DEP will be integrated as a feature into a Rehydration Calculator application that has previously been tested as part of two clinical studies in Bangladesh (icddr,b/ IEDCR; Khan AI et al, submitted 2019 and Haque et al. PLoS NTD 2017). The Rehydration Calculator with the DEP turned 'off' will serve as a control. The experimental arm of the study will have the DEP turned 'on'. The Rehydration Calculator is an electronic decision-support tool that provides recommendations for rehydration based on entry of age, gender, weight, and dehydration status. Given the outcome measures of this study, the Rehydration Calculator will be configured to not give antibiotic recommendations.

Study Sites: 4 Healthcare Facilities in Mali. (CSRef CV, ASACOSAB1, CSRef CVI, ASACOYIR)

Clinical setting:
The clinical approach will adhere to World Health Organization guidelines for the management of uncomplicated diarrhoeal disease in children with No, Some and Severe dehydration. These guidelines have previously been adapted to a digital format (e.g. Rehydration Calculator) and evaluated in a pilot and cluster randomized controlled trial (icddr,b/ IEDCR; Khan AI et al, submitted 2019 and Haque et al. PLoS NTD 2017); clinically relevant outcome measures in these studies were equivalent or improved with digital decision-support. The Rehydration Calculator version to be used in this study will include unchanged weights estimations and oral and intravenous (IV) fluid calculations; changes will include elimination of the antibiotic recommendations and replacement with the viral diarrhea probability. 15 physicians across 4 facilities will be enrolled.

Operational Responsibilities:

All participant-based research, including consents, will be conducted in Mali. The DEP algorithm will be integrated as a feature into a Rehydration Calculator application that has previously been tested as part of two clinical studies in Bangladesh (icddr,b/ IEDCR; Khan AI et al, submitted 2019) and Haque et al. PLoS NTD 2017). The Rehydration Calculator with the DEP turned 'off' will serve as a control. The experimental arm of the study will have the DEP turned 'on'. The Rehydration Calculator is an electronic decision support tool that provides recommendations for rehydration based on entry of age, gender, weight, and dehydration status. Given the outcome measures of this study, the Rehydration Calculator will be configured to not give antibiotic recommendations.

a. Detailed Research Methods

c.i. **Study design.**

This is a randomized crossover study, where clinicians will be randomized to periods where they will use a rehydration calculator application with or without the DEP algorithm. The crossover will include a washout period to reduce carryover effect. The study will be conducted over a 9-week period. We will use a random number generator to randomize clinicians to DEP (use of the etiology calculator) or control arm (use of a previously-tested rehydration calculator) within site for the first 4 weeks. After the first 4 weeks, there will be 1-week washout period without decision support, after which each clinician will cross-over to the other arm for the next 4 weeks.

c.ii. **Study population and enrolment:**

We will enrol both 1) diarrhea-treating clinicians, and 2) children presenting with acute diarrhea.

We will use the following inclusion/exclusion criteria for enrolling clinicians:

Inclusion criteria:

- Physician providing acute care for children with diarrhea at study hospitals
- Available to answer survey questionnaire

Exclusion criteria:

- Planning to leave the study site prior to completion of the research
- Inability to read

We will utilize the following inclusion/exclusion criteria when enrolling children:

Inclusion criteria:

- Age 2-59 months
- Less than 7 days of diarrheal symptoms from onset to presentation
- Three or more loose stools in the prior 24 hours
- Access to use of a cell phone

Exclusion criteria:

- No parent or primary caretaker available for consent
- Diarrhea lasting longer than 7 days
- Severe pneumonia, severe sepsis, meningitis, or other condition aside from gastroenteritis
- MUAC of <=115 if >6 months, or <=110 if 2 to 6 months of age [we will exclude severely malnourished children because they would follow a different antibiotic protocol]

For the purpose of enrolment in our study, we will utilize the World Health Organization (WHO) definition for acute diarrhea, which is 3 or more loose stools per day in the last 24 hours. Subjects with chronic diarrhea (diarrhea lasting more than 7 days) will be excluded, as this study is focused on acute diarrhea.

Staff Training: Staff will receive intensive training regarding all study procedures prior to the study. A study coordinator will be trained to use the randomization block and distribute appropriate mobile phones to physicians and keep track of the devices. The study coordinator will also be in charge of administering questionnaires to physicians (details described in data collection section).

Staff will enrol patients according to the inclusion/exclusion criteria and they will receive training which includes an in-depth review of how to appropriately assess symptoms and MUAC. They will also be trained in calling patients for a post-visit follow up questionnaire.

Doctors will receive training on how to use the Rehydration Calculator and DEP application prior to the study. They will complete questionnaires before and after the study regarding previous use of clinical calculators, access to internet and willingness to use a new app that will help to predict etiology of diarrhea.

Enrolment and consent of physicians

At each site, we will recruit clinicians whose responsibilities include the care of paediatric diarrhea patients. Study staff will determine if the clinician is eligible and then provide detailed information of the study. Once the clinician exhibits understanding, written consent will be obtained. There will be two questionnaires administered: 1) before, and 2) after the study period. The post-study questionnaire will also include a brief focused interview. Only clinicians who have completed the entire study will be eligible for this questionnaire.

Enrolment and consent of patients

Study staff will be situated in the intake areas and will identify children 2–59 months with diarrhoeal illness. Study staff will select consecutive subjects between 8am and 2pm on weekdays for enrolment on arrival to the Hospital. Potential participants' information (age and time of intake, etc) will be recorded in a screening log. Study staff and nurses will inform parents or primary caretakers (PPC) of patients who meet the inclusion/exclusion criteria about the study. If PPC is interested in the study, study staff will determine if the child is eligible and then provide more detailed information to the PPC's of eligible subjects. Detailed information will be provided in French and will include goals, risks, and benefits of the study. Once the PPC exhibits understanding, written consent will be obtained in the local language prior to enrolling the subject in the study. Illiterate PPC's will give consent by marking the consent form with their thumbprint or signature after a study staff member reads the consent form in French. Children without a PPC present will be excluded from the study. The last signature on the consent will be from the enrolling staff member.

c.iii. Data collection

Clinicians: We will use a screening form (Appendix 3) and questionnaire (Pre- and post-Study) to collect quantitative (Appendix 4 & 5) data regarding clinician attitudes and experiences using the App and its role in diarrheal management. These questionnaires will be administered by study coordinators at 2 time points: 1) at the start of the study, and 2) at the end of the study. We will also use the App itself to collect data regarding physician usage.

Children: For each enrolled patient, study staff will collect:

- At enrolment, demographic and clinical variables
- At discharge, hospital outcome measures, including intravenous and oral fluids, and antibiotics, zinc administered
- At 10-days post-discharge, health outcomes including persistence of diarrheal and abdominal symptoms, returns to healthcare, and medication use since discharge, will be collected over a phone call.

Instruments: As mentioned, electronic tools that are compliant to the protections of PHI (Protected Health Information) will be used in this study. These include the Outbreak Responder software, which includes the DEP, and if needed, RedCap.

Data Storage: Data collected on Outbreak Responder software will be stored on a secure cloud-based server (server is physically based in Singapore; this is a HIPAA compliant server managed by Amazon Web Services).

Access to the server(s) will be restricted and the server management conforms to industry standard for health information.

Sample Size Calculation and Outcome (Primary and Secondary) Variable(s)

For estimated 90% of children receiving antibiotics in control arm (per mHDM study), and estimated 80% of children receiving antibiotics in DEP arm, along with a within- and between- period correlations for each physician of 0.15, a sample size of 360 children (**15 clinicians**, with 2 cross-over periods and 12 patients during each period) will achieve a power above 95% to detect a 2-sided significance of 0.05. If enrolment is reached prior to the pre-determined study period, enrolment will be continued until the end of the study period; this is important given that aspects of the study outcomes are dependent on weeks of use.

Data Analysis

The primary endpoint of estimating the proportion difference of children given antibiotic prescriptions in the control versus the DEP arms can be analyzed using a generalized linear mixed model (GLMM). The response is binary (yes or no), and we will treat provider and clinic as random, while accounting for the time period in order to estimate the treatment effect. All data analyses will be conducted in R. For the physician satisfaction end point, we will use characteristic tables to summarize the results of the survey.

Data Safety Monitoring Plan (DSMP)

A Data and Safety Monitoring Plan (DSMP) will be made for the study. It will provide the overall framework for the research protocol's data and safety monitoring. Adverse events (anticipated or unanticipated) will be reported to the DSMB/ERC within 24 hours for severe adverse events (SAE), with independent reports submitted to all participating DSMB committees.

Direct Access to Source Data/Documents

The investigator/institutions will permit (by way of written agreement) trial-related monitoring, audits, DSMB review, and regulatory inspection; providing direct access to source data/documents.

Assessment of Safety

All adverse events which are observed actively or reported/volunteered by subject/guardian will be recorded with information about severity (i.e., whether mild, moderate or severe) and possible relation to the study intervention by the study physician. In case of any serious adverse events, the investigator will notify DSMB immediately by telephone/facsimile/email within 24 hrs and take appropriate measures to safeguard the participants. Every SAE must be reported, even if the Investigator considers that it is not related to the intervention. The Completed serious adverse event (SAE) form should be submitted to DSMB within 72 hours either by fax, or by email.

Definitions of Serious Adverse Events (SAE) and Adverse Events:

Serious Adverse Event: Any adverse event that results any of the following outcomes: death, significant disability/incapacity, and/or life-threatening situation of any patient within the study period (enrolment of patient at admission to 10 days after discharge).

Adverse Events: An adverse event will be defined as an untoward medical event temporally associated with a medical intervention in a patient that does not necessarily have a causal relationship with the treatment.

Definitions to guide causal relationship SAE:

Very Likely/Certain:	A clinical event with a plausible time relationship to intervention and which cannot be explained by concurrent disease or other drugs or chemicals.
Probable:	A clinical event with a reasonable time relationship to intervention; is unlikely to be attributed to concurrent disease or other drugs or chemicals.
Possible:	A clinical event with a reasonable time relationship to intervention, but which could also be explained by concurrent disease or other drugs or chemicals.

Unlikely:	A clinical event whose time relationship to intervention makes a causal connection improbable, but which could be plausibly explained by underlying disease or other drugs or chemicals
Unrelated:	A clinical event with an incompatible time relationship and which could be explained by underlying disease or other drugs or chemicals.
Unclassifiable**:**	A clinical event with insufficient information to permit assessment and identification of the cause.

Ethical Assurance for Protection of Human rights

Justification of conducting research in human participants that are sick: The questions asked herein can only be studied in humans because we are interested in how best to develop a DEP algorithm for humans to improve clinical care.

Benefits: Patients will likely have no immediate benefit. All populations at risk from diarrhoeal disease will benefit by the knowledge gained and development of an DEP.

Risks*:* We are comparing the effect of a DEP on antibiotic orders in the context of WHO guidelines. These guidelines and calculations have been extensively reviewed and tested in previous studies at CVD-Mali. Despite these efforts, patients will remain at minor risk that technical errors may occur in the DEP. To minimize this risk, the DEP has been tested in a pilot study in Mali.

Protections of participant human rights.

Clinical. Standard of care for the treatment of patients with diarrhoeal disease will be maintained throughout this study. There will be no differential treatment to specific genders, age groups, races, and cultures. There are also no special procedures or invasive procedures included in this study. Patient histories taken during patient care directly related to the demographic and clinical information are required to treat the patient and identify public health interventions that will benefit the community (e.g. diarrhoeal disease cluster analysis).

Confidentiality. One risk is a breach in confidentiality (see privacy protection sections). In brief, to address this risk, data collected via the application will be stored on a secure, password protected computer interface. No data will be stored permanently on mobile devices. Data will be transiently stored on the devices when there is no mobile connectivity. All devices will be password protected and encryption occurs at each point in the software architecture. Access to the server will be restricted, and when possible, data will be coded before analysis. Data analysis performed in the United States will be stored on a local secure, HIPAA compliant server. All paper documents will be stored in a locked container with limited access governed by the study PI.

Individual privacy.

Privacy protections: Diarrhoeal disease patients need to be assessed rapidly in hospital triage setting. Although there is limited time and space to assure complete privacy, staff maintain appropriate protocols for the protection of privacy during the triage and admission process.

PHI confidentiality: Health information will include: demographic data (e.g. name, birth date, residence location, hospital location, telephone number), clinical data (e.g. number of stools, watery, bloody, vomiting, medications taken for the illness, allergies), and biometric data (weight and MUAC). PHI institutional regulations will be followed. When there is a need for US-based staff to work with PHI or import PHI into the United States, staff will comply by US NIH HIPAA based regulations.

Access to the data: Access to the data is based on levels of access: (i) Collection of clinical information by the research staff and (ii) Access to the database (PHI): core-research team and named key personnel in the (USA).

Education of staff: We will assure that all staff takes personal health information privacy training.

Safe-gaurds: Physical: As mentioned, standard of care will be performed during this study. Oversight will come immediately by the Senior Field Assistant (Trained Field Assistant) who will report to the Study Doctor.

Financial: There is no cost to participate in the study.

Social concerns: Unanticipated social concerns will be evaluated in partnership and alignment meetings across all collaborators and local hospital staff/leaders.

Conflict of interest: The study investigators and affiliates hold NO patents or stock in the decision-support technologies.

Collaborative Arrangements

Data sharing. The data ownership, access and security of personal health information related to this study will abide by CVD-Mali policies. These policies will be honoured by University of Utah, University of Maryland, and CVD-Mali investigators. For these reasons, primary access to the data will be governed by Dr. Samba Sow (CVD-Mali PI).

Intellectual Property. The study investigators and affiliates hold NO patents or stock in the decision-support technologies. The algorithms used in the software are derived from publicly available WHO protocols. Therefore, there is no intellectual property to be secured in regards to the decision support because these are published protocols.

Authorship. Authorship will be granted based on CVD-Mali, University of Utah and University of Maryland policies. In general, all members of the team that contribute essential materials, significant contributions to experimental design, implementation, and data analysis, and participate in the writing manuscripts will be offered authorship.

Sample collection. Not applicable.

Literature Cited

1. Global Burden of Disease Pediatrics C, Kyu HH, Pinho C, Wagner JA, Brown JC, Bertozzi-Villa A, Charlson FJ, Coffeng LE, Dandona L, Erskine HE, Ferrari AJ, Fitzmaurice C, Fleming TD, Forouzanfar MH, Graetz N, Guinovart C, Haagsma J, Higashi H, Kassebaum NJ, Larson HJ, Lim SS, Mokdad AH, Moradi-Lakeh M, Odell SV, Roth GA, Serina PT, Stanaway JD, Misganaw A, Whiteford HA, Wolock TM, Wulf Hanson S, Abd-Allah F, Abera SF, Abu-Raddad LJ, AlBuhairan FS, Amare AT, Antonio CA, Artaman A, Barker-Collo SL, Barrero LH, Benjet C, Bensenor IM, Bhutta ZA, Bikbov B, Brazinova A, Campos-Nonato I, Castaneda-Orjuela CA, Catala-Lopez F, Chowdhury R, Cooper C, Crump JA, Dandona R, Degenhardt L, Dellavalle RP, Dharmaratne SD, Faraon EJ, Feigin VL, Furst T, Geleijnse JM, Gessner BD, Gibney KB, Goto A, Gunnell D, Hankey GJ, Hay RJ, Hornberger JC, Hosgood HD, Hu G, Jacobsen KH, Jayaraman SP, Jeemon P, Jonas JB, Karch A, Kim D, Kim S, Kokubo Y, Kuate Defo B, Kucuk Bicer B, Kumar GA, Larsson A, Leasher JL, Leung R, Li Y, Lipshultz SE, Lopez AD, Lotufo PA, Lunevicius R, Lyons RA, Majdan M, Malekzadeh R, Mashal T, Mason-Jones AJ, Melaku YA, Memish ZA, Mendoza W, Miller TR, Mock CN, Murray J, Nolte S, Oh IH, et al., 2016. Global and National Burden of Diseases and Injuries Among Children and Adolescents Between 1990 and 2013: Findings From the Global Burden of Disease 2013 Study. JAMA Pediatr 170: 267-87.
2. Niehaus MD, Moore SR, Patrick PD, Derr LL, Lorntz B, Lima AA, Guerrant RL, 2002. Early childhood diarrhoea is associated with diminished cognitive function 4 to 7 years later in children in a northeast Brazilian shantytown. Am J Trop Med Hyg 66: 590-3.
3. Shane AL, Mody RK, Crump JA, Tarr PI, Steiner TS, Kotloff K, Langley JM, Wanke C, Warren CA, Cheng AC, Cantey J, Pickering LK, 2017. 2017 Infectious Diseases Society of America Clinical Practice Guidelines for the Diagnosis and Management of Infectious Diarrhoea. Clin Infect Dis.
4. Reilly BM, Evans AT, 2006. Translating clinical research into clinical practice: impact of using prediction rules to make decisions. Ann Intern Med 144: 201-9.
5. Ban JW, Emparanza JI, Urreta I, Burls A, 2016. Design Characteristics Influence Performance of Clinical Prediction Rules in Validation: A Meta-Epidemiological Study. PLoS One 11: e0145779.
6. Maguire JL, Kulik DM, Laupacis A, Kuppermann N, Uleryk EM, Parkin PC, 2011. Clinical prediction rules for children: a systematic review. Pediatrics 128: e666-77.
7. Gorelick MH, Shaw KN, Murphy KO, 1997. Validity and reliability of clinical signs in the diagnosis of dehydration in children. Pediatrics 99: E6.
8. Bailey B, Gravel J, Goldman RD, Friedman JN, Parkin PC, 2010. External validation of the clinical dehydration scale for children with acute gastroenteritis. Acad Emerg Med 17: 583-8.
9. 1996. Practice parameter: the management of acute gastroenteritis in young children. American Academy of Pediatrics, Provisional Committee on Quality Improvement, Subcommittee on Acute Gastroenteritis. Pediatrics 97: 424-35.
10. Armon K, Stephenson T, MacFaul R, Eccleston P, Werneke U, 2001. An evidence and consensus based guideline for acute diarrhoea management. Arch Dis Child 85: 132-42.
11. Levine AC, Munyaneza RM, Glavis-Bloom J, Redditt V, Cockrell HC, Kalimba B, Kabemba V, Musavuli J, Gakwerere M, Umurungi JP, Shah SP, Drobac PC, 2013. Prediction of severe disease in children with diarrhoea in a resource-limited setting. PLoS One 8: e82386.
12. DeWitt TG, Humphrey KF, McCarthy P, 1985. Clinical predictors of acute bacterial diarrhoea in young children. Pediatrics 76: 551-6.
13. Fontana M, Zuin G, Paccagnini S, Ceriani R, Quaranta S, Villa M, Principi N, 1987. Simple clinical score and laboratory-based method to predict bacterial etiology of acute diarrhoea in childhood. Pediatr Infect Dis J 6: 1088-91.
14. Klein EJ, Boster DR, Stapp JR, Wells JG, Qin X, Clausen CR, Swerdlow DL, Braden CR, Tarr PI, 2006. Diarrhoea etiology in a Children's Hospital Emergency Department: a prospective cohort study. Clin Infect Dis 43: 807-13.
15. Pavlinac PB, Denno DM, John-Stewart GC, Onchiri FM, Naulikha JM, Odundo EA, Hulseberg CE, Singa BO, Manhart LE, Walson JL, 2016. Failure of Syndrome-Based Diarrhoea Management Guidelines to Detect Shigella Infections in Kenyan Children. J Pediatric Infect Dis Soc 5: 366-374.
16. Kotwani A, Chaudhury RR, Holloway K, 2012. Antibiotic-prescribing practices of primary care prescribers for acute diarrhoea in New Delhi, India. Value Health 15: S116-9.
17. Osatakul S, Puetpaiboon A, 2007. Appropriate use of empirical antibiotics in acute diarrhoea: a cross-sectional survey in southern Thailand. Ann Trop Paediatr 27: 115-22.

18. Pathak D, Pathak A, Marrone G, Diwan V, Lundborg CS, 2011. Adherence to treatment guidelines for acute diarrhoea in children up to 12 years in Ujjain, India--a cross-sectional prescription analysis. BMC Infect Dis 11: 32.
19. Kotloff KL, Nataro JP, Blackwelder WC, Nasrin D, Farag TH, Panchalingam S, Wu Y, Sow SO, Sur D, Breiman RF, Faruque AS, Zaidi AK, Saha D, Alonso PL, Tamboura B, Sanogo D, Onwuchekwa U, Manna B, Ramamurthy T, Kanungo S, Ochieng JB, Omore R, Oundo JO, Hossain A, Das SK, Ahmed S, Qureshi S, Quadri F, Adegbola RA, Antonio M, Hossain MJ, Akinsola A, Mandomando I, Nhampossa T, Acacio S, Biswas K, O'Reilly CE, Mintz ED, Berkeley LY, Muhsen K, Sommerfelt H, Robins-Browne RM, Levine MM, 2013. Burden and aetiology of diarrhoeal disease in infants and young children in developing countries (the Global Enteric Multicenter Study, GEMS): a prospective, case-control study. Lancet 382: 209-22.
20. Adams ST, Leveson SH, 2012. Clinical prediction rules. BMJ 344: d8312.
21. Gage BF, Waterman AD, Shannon W, Boechler M, Rich MW, Radford MJ, 2001. Validation of clinical classification schemes for predicting stroke: results from the National Registry of Atrial Fibrillation. JAMA 285: 2864-70.
22. Antman EM, Cohen M, Bernink PJ, McCabe CH, Horacek T, Papuchis G, Mautner B, Corbalan R, Radley D, Braunwald E, 2000. The TIMI risk score for unstable angina/non-ST elevation MI: A method for prognostication and therapeutic decision making. JAMA 284: 835-42.
23. Jones BE, Jones J, Bewick T, Lim WS, Aronsky D, Brown SM, Boersma WG, van der Eerden MM, Dean NC, 2011. CURB-65 pneumonia severity assessment adapted for electronic decision support. Chest 140: 156-163.
24. Wallace E, Uijen MJ, Clyne B, Zarabzadeh A, Keogh C, Galvin R, Smith SM, Fahey T, 2016. Impact analysis studies of clinical prediction rules relevant to primary care: a systematic review. BMJ Open 6: e009957.
25. Bright TJ, Wong A, Dhurjati R, Bristow E, Bastian L, Coeytaux RR, Samsa G, Hasselblad V, Williams JW, Musty MD, Wing L, Kendrick AS, Sanders GD, Lobach D, 2012. Effect of clinical decision-support systems: a systematic review. Ann Intern Med 157: 29-43.
26. McGinn TG, McCullagh L, Kannry J, Knaus M, Sofianou A, Wisnivesky JP, Mann DM, 2013. Efficacy of an evidence-based clinical decision support in primary care practices: a randomized clinical trial. JAMA Intern Med 173: 1584-91.
27. Worrall G, Hutchinson J, Sherman G, Griffiths J, 2007. Diagnosing streptococcal sore throat in adults: randomized controlled trial of in-office aids. Can Fam Physician 53: 666-71.

28. Paul, R.C., *et al.* Incidence of severe diarrhoea due to Vibrio cholerae in the catchment area of six surveillance hospitals in Bangladesh. *Epidemiol Infect*, 1-13 (2015).
