# Supplementary material for: Improving Antibiotic Stewardship for Diarrheal Disease With Probability-Based Electronic Clinical Decision Support: A Randomized Crossover Trial
Source: JAMA Pediatr. 2022 Aug 29;176(10):973–9. doi: 10.1001/jamapediatrics.2022.2535 (PMC9425282; doi:10.1001/jamapediatrics.2022.2535)
Supplement: Supplement 4. — Data sharing statement [file jamapediatr-e222535-s004.pdf]

Data Sharing Statement

Nelson. Improving Antibiotic Stewardship for Diarrheal Disease With Probability-Based Electronic Clinical Decision Support. *JAMA Pediatr*. Published August 29, 2022. doi:10.1001/jamapediatrics.2022.2535

Data
Data available: Yes
Data types: Deidentified participant data, Data dictionary
How to access data: University of Utah Repository: https://github.com/LeungLab/DEPcrossover
When available: With publication

Supporting Documents
Document types: Statistical/analytic code
How to access documents: University of Utah Repository: https://github.com/LeungLab/DEPcrossover
When available: With publication

Additional Information
Who can access the data: Anyone requesting the data.
Types of analyses: For any purpose or for a specified purpose.
Mechanisms of data availability: Without investigator support.
Any additional restrictions: None.
